# Bicycle-Related Acetabular Fractures Are Associated with Anterior Column Involvement: A Multicenter Study with an Exploratory Analysis of Clipless Pedal Use

**DOI:** 10.3390/jcm15207918

**Published:** 2026-10-14

**Authors:** Bong-Ju Lee, Hwan-Hee Lee, Sung-Min Kim, Pichayut Wattanapreechanon, Se-Won Lee

**Affiliations:** 1 Department of Orthopedic Surgery, Daejeon Sun Hospital, Daejeon 34811, Republic of Korea; bonjoolee@hanmail.net; 2 Department of Orthopedic Surgery, Graduate School, The Catholic University of Korea, Seoul 06591, Republic of Korea; 3 Department of Orthopedic Surgery, Daejeon St. Mary’s Hospital, College of Medicine, The Catholic University of Korea, Daejeon 34943, Republic of Korea; hwanhee337@naver.com; 4 Department of Orthopedic Surgery, Yeouido St. Mary’s Hospital, College of Medicine, The Catholic University of Korea, 10 63-ro, Yeongdeungpo-gu, Seoul 07345, Republic of Korea; gatetempler@naver.com (S.-M.K.); pichayut@nmu.ac.th (P.W.); 5 Department of Orthopedics, Faculty of Medicine Vajira Hospital, Navamindradhiraj University, 3 Thanon Khao, Wachira Phayaban, Dusit District, Bangkok 10300, Thailand

**Keywords:** acetabular fracture, bicycle accident, cycling injury, clipless pedal, cleat shoes, anterior column, Judet–Letournel classification, clinical data warehouse

## Abstract

**Background/Objectives**: Cycling-related acetabular fractures have not been well described. We aimed to determine the proportion of acetabular fractures associated with bicycle accidents and to compare their fracture patterns with those caused by other mechanisms. Findings related to clipless pedal use were considered exploratory. **Methods**: Patients treated for an acetabular fracture between January 2010 and December 2023 were identified by ICD-10 codes from a university hospital network Clinical Data Warehouse. After exclusions, 378 patients with 385 fractures were classified according to the Judet–Letournel system. Fracture-pattern proportions were compared using Fisher’s exact test, and the association with anterior column involvement was examined with logistic regression adjusted for age and sex. Cleat-shoe status, obtained from medical records or telephone interviews, was analyzed descriptively. **Results**: Bicycle accidents were the mechanism of injury in 26 of 378 patients (6.9%). Compared with fractures caused by other mechanisms, bicycle-related fractures showed a higher proportion of pure anterior column fractures (38.5% versus 16.7%, *p* = 0.014) and anterior column involvement (65.4% versus 38.2%, *p* = 0.011). The association with anterior column involvement remained after adjustment for age and sex (adjusted odds ratio 3.02, 95% CI 1.31–6.98). Among 12 confirmed cleat-shoe users, 7 (58.3%) had a pure anterior column fracture, 10 (83.3%) had anterior column involvement, and 8 (66.7%) had extension into the acetabular roof. **Conclusions**: Bicycle-related acetabular fractures were associated with a higher proportion of anterior column involvement, and the association remained after adjustment for age and sex. Because bone quality and injury energy could not be adjusted for, this association should not be interpreted as causal. The anterior-dominant pattern among confirmed cleat-shoe users is exploratory and should not be interpreted causally.

## 1. Introduction

Cycling has steadily increased in popularity over recent decades, both as a mode of transport and as a recreational and competitive sport. Traumatic injuries caused by falls and collisions have therefore become more frequent. Large cycling-injury cohorts have shown that fractures involve mainly the upper extremity, and that pelvic and acetabular injuries are less common [1,2]. A recent Swedish Fracture Register study of 33,384 bicycle-related fractures identified only 179 acetabular fractures (0.5% of all fractures). That study confirmed that these injuries occur within the broad spectrum of cycling fractures, but it did not describe acetabular fracture morphology and did not determine the proportion of all acetabular fractures that is attributable to bicycle accidents [2].

Few studies have focused specifically on cycling-related acetabular fractures. Swart et al. reported four patients who sustained an acetabular fracture after a cycling-related lateral fall, and they emphasized that these fractures may be missed on plain radiographs. All four fractures involved the anterior column [1]. Parker et al. described a displaced intracapsular femoral neck fracture in a 38-year-old club cyclist who was unable to release his foot from a clipless pedal and fell directly onto the hip [3]. These reports suggest that a lateral bicycle fall can transmit a substantial force to the hip region. However, they do not indicate how often bicycle accidents cause acetabular fractures or whether these fractures have a characteristic morphological pattern. Knowing the morphological distribution is also clinically relevant, because the fracture pattern guides the surgical approach and the fixation strategy [4,5].

**Figure 1:**
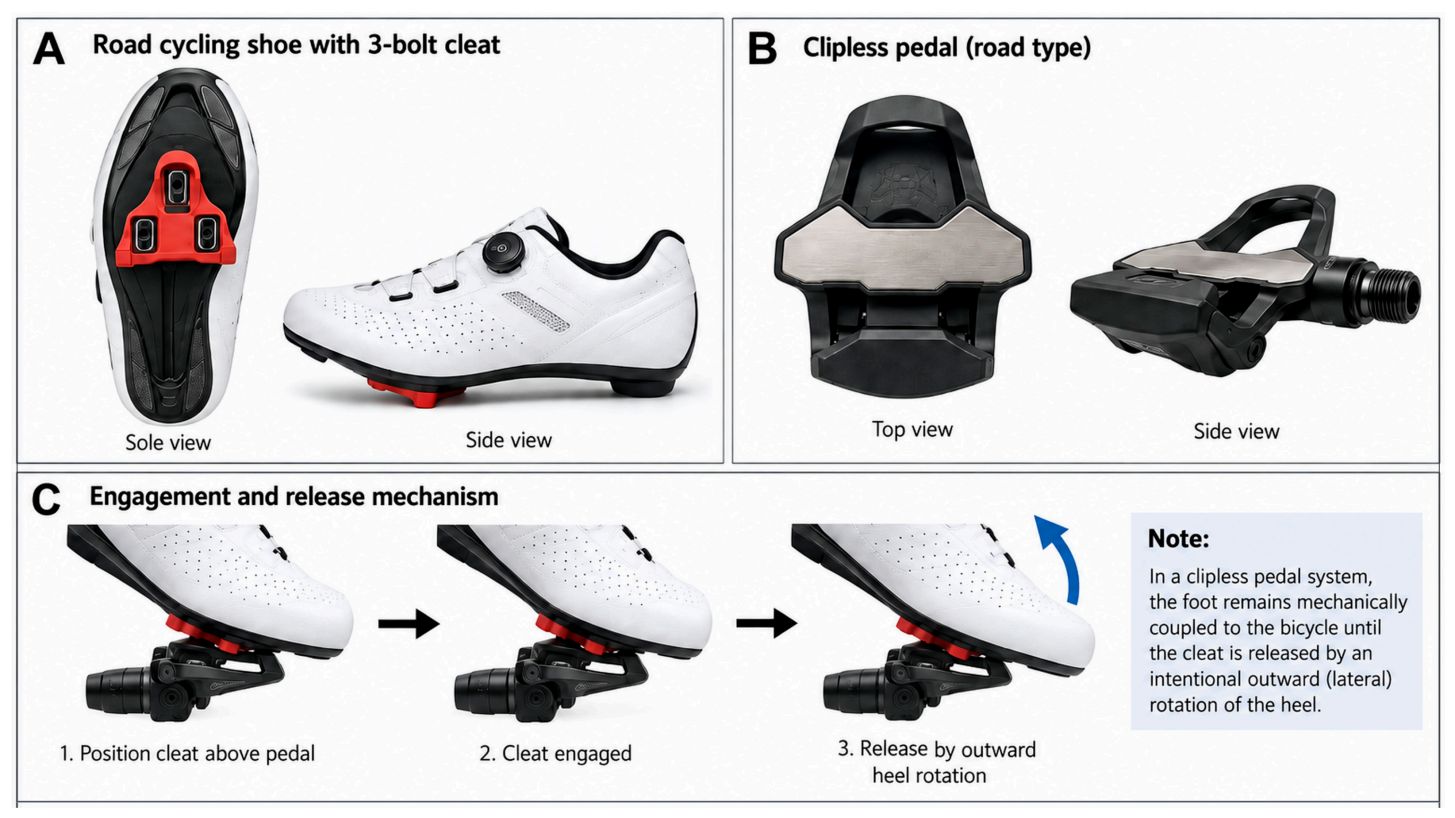
Clipless pedal system and the mechanism of engagement and release. (**A**) Road cycling shoe with a three-bolt cleat. (**B**) Corresponding clipless pedal of the road type. (**C**) Sequence of engagement and release: the cleat is secured to the pedal by downward pressure and is released by an outward rotation of the heel. The illustration is a conceptual schematic and does not depict any specific commercial product. It was created with the assistance of a generative artificial intelligence tool and was reviewed and edited by the authors (see Section 2.6).

Two points may be relevant. First, the modern cleat pedal system, which is also called the clipless pedal system, mechanically couples the cycling shoe to the pedal. If a rider fails to disengage in time, the foot may remain attached to the pedal. In this situation, the rider may not be able to place the foot on the ground for protection, and the opportunity to reduce the impact before the lateral aspect of the hip strikes the ground may be decreased. Second, a lateral fall with direct impact on the greater trochanter is a recognized mechanism of low-energy acetabular fracture in older adults. In geriatric patients, the force is transmitted from the greater trochanter through the femoral neck and head toward the anterior and superior acetabulum, and the anterior structures are frequently involved [6,7]. A similar force pathway may contribute to the morphology of some cycling-related fractures, especially when the lower limb remains constrained during the fall. The construction of the clipless pedal system and the sequence of engagement and release are shown in Figure 1.

The primary purpose of the present study was to determine the proportion of acetabular fractures associated with bicycle accidents in a large multicenter cohort and to compare the fracture patterns of bicycle-related fractures with those caused by other mechanisms. A secondary, exploratory objective was to describe the fracture morphology observed among riders confirmed to have been wearing cleat shoes at the time of injury.

## 2. Materials and Methods

### 2.1. Study Design and Ethical Approval

This was a retrospective, multicenter, observational study. The study was approved by the Institutional Review Board of Daejeon St. Mary’s Hospital, College of Medicine, The Catholic University of Korea (approval number DC24WISI0008, approved on 6 February 2024). The requirement for written informed consent was waived by the Institutional Review Board because of the retrospective design of the study. Verbal consent was obtained from patients who participated in the telephone interview described in Section 2.3. The study was conducted in accordance with the Declaration of Helsinki.

### 2.2. Data Source and Patient Selection

Patients were identified through the Clinical Data Warehouse of the Catholic Medical Center. The Catholic Medical Center has operated this data warehouse since November 2019 as a single platform that integrates the electronic medical records of its eight affiliated university hospitals. It holds the demographic data of approximately 15 million patients together with their diagnoses coded by ICD-10, narrative reports, medications, laboratory results and radiological records, and data are provided to researchers through an institutional data-utilization review and approval by the Institutional Review Board [8]. The same platform has been used for multicenter retrospective studies that draw on the affiliated hospitals simultaneously [9]. For the present study, the records of six of the eight affiliated hospitals were searched. Because bicycle-related acetabular fractures are uncommon, a multicenter database was used to obtain a sufficient number of cases and an appropriate denominator. The following ICD-10 diagnostic codes were used for the initial search: S32.3 (fracture of ilium), S32.4 (fracture of acetabulum), S32.7 (multiple fractures of lumbar spine and pelvis), and S32.8 (fracture of other and unspecified parts of lumbar spine and pelvis). The search period was from 1 January 2010 to 31 December 2023, a period of 14 years.

**Figure 2:**
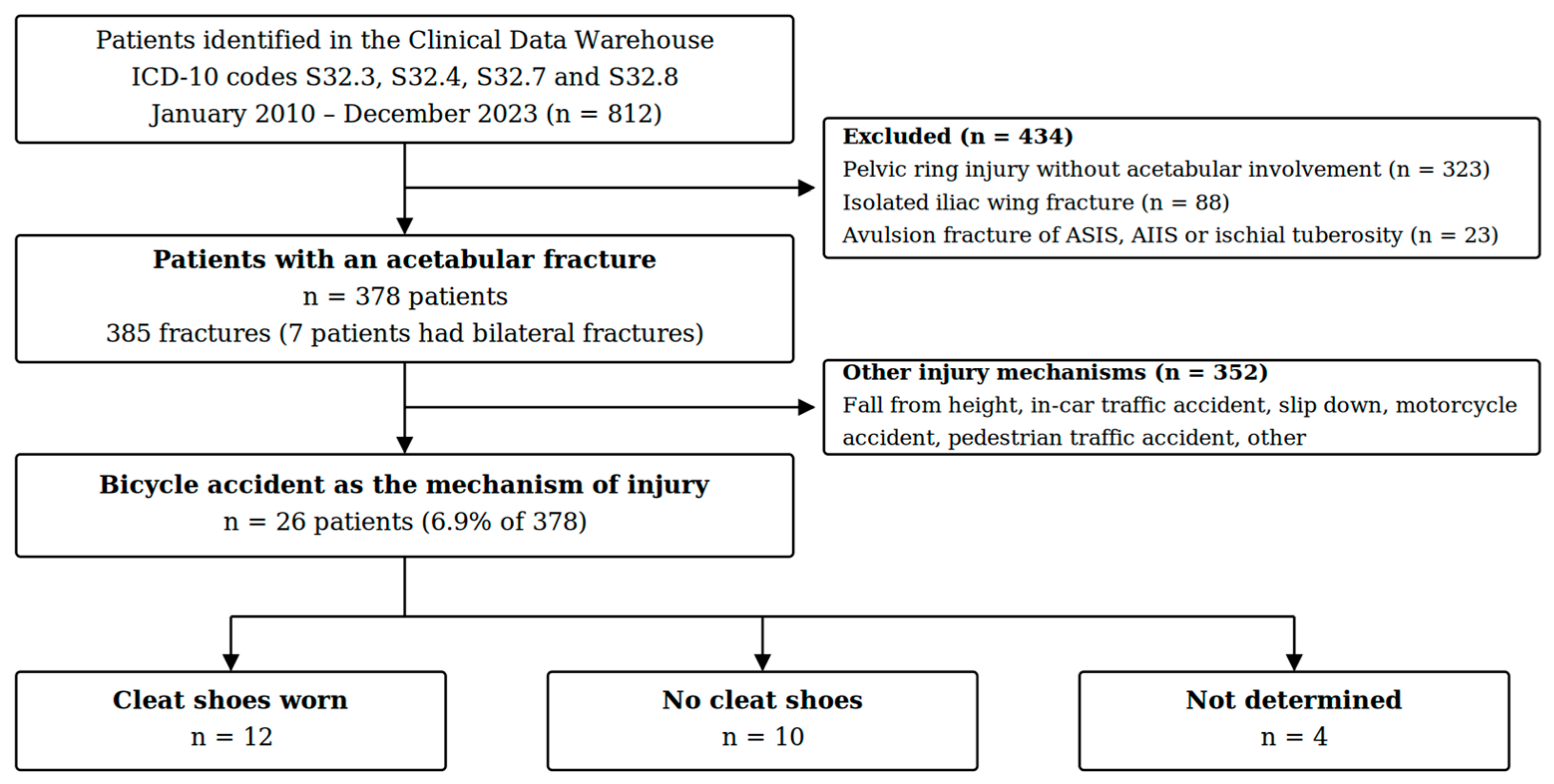
Flow diagram of patient selection. A total of 812 patients were identified from the Clinical Data Warehouse using ICD-10 codes S32.3, S32.4, S32.7, and S32.8 for the period from January 2010 to December 2023. After exclusion of 323 patients with a pelvic ring injury, 88 patients with an isolated iliac wing fracture, and 23 patients with an avulsion fracture, 378 patients with 385 acetabular fractures were included. Of these, 26 patients sustained the injury in a bicycle accident, and 12 of these were wearing cleat shoes at the time of injury.

The initial search retrieved 812 patients. The medical records and the admission imaging studies of all 812 patients were reviewed, and the following patients were excluded: 323 patients with a pelvic ring injury without acetabular involvement, 88 patients with an isolated iliac wing fracture, and 23 patients with an avulsion fracture of the anterior superior iliac spine, the anterior inferior iliac spine, or the ischial tuberosity. After these exclusions, 378 patients remained. Seven of these patients had bilateral acetabular fractures, so that a total of 385 acetabular fractures were available for classification (Figure 2).

### 2.3. Data Collection

The following variables were collected from the medical records: age at the time of injury, sex, the side of the injured acetabulum, the mechanism of injury, and whether the patient was treated operatively or non-operatively.

The mechanism of injury was categorized as fall from height, in-car traffic accident, slip down, motorcycle accident, pedestrian traffic accident, bicycle accident, or other. For patients whose mechanism was a bicycle accident, it was additionally recorded whether the accident involved a collision with another vehicle and whether the rider was wearing cleat shoes at the time of injury.

Cleat-shoe use was first assessed from the medical records. For patients whose injury was confirmed as a bicycle accident but whose cleat-shoe use was not documented, an additional telephone interview regarding the circumstances of the injury was conducted between March 2024 and July 2024. Patients who could not be contacted or who declined to answer were classified as undetermined, and four patients fell into this category.

### 2.4. Radiographic Evaluation

All fractures were classified according to the Judet–Letournel classification [10] using the plain radiographs and the computed tomography images obtained at the time of admission. The six participating hospitals are affiliated hospitals of the same medical college and share a common picture archiving and communication system, so that the original images of all patients could be reviewed centrally. At all six hospitals, the standard imaging for a suspected acetabular fracture consisted of an anteroposterior radiograph of the pelvis and a computed tomography scan of the pelvis, and both were used for the classification. The classification was performed by one orthopedic surgeon (B.-J.L., 19 years of experience in orthopedic surgery) and was verified for every fracture by a second orthopedic surgeon (S.-W.L., 20 years of experience, specialized in pelvic and acetabular trauma). Fractures on which the two surgeons disagreed were reviewed together, and the consensus classification was used. The fractures were not classified independently, and interobserver agreement was not measured. The ten categories of the classification were used, namely anterior wall, anterior column, posterior wall, posterior column, posterior column with posterior wall, transverse, transverse with posterior wall, T-shaped, anterior column with posterior hemitransverse, and both columns.

For the analysis, “pure anterior column fracture” refers to the anterior column category alone. “Anterior column involvement” was defined in advance as the sum of the anterior column, anterior column with posterior hemitransverse, and both column categories, because in each of these three patterns the anterior column is a structurally involved component.

In the subgroup of patients who were wearing cleat shoes, the following additional morphological features were recorded from the admission imaging studies: extension of the fracture line into the acetabular roof, extension of the fracture line to the level of the iliopubic eminence, impaction of the superior dome, the presence of a posterior hemitransverse component, and a both column pattern. These features were not mutually exclusive, and more than one feature could be present in the same fracture.

### 2.5. Statistical Analysis

Continuous variables are presented as the mean with the standard deviation and the range, and categorical variables as the number and percentage. Because several cell counts were small, proportions were compared using two-sided Fisher’s exact tests rather than chi-square tests. Odds ratios and 95% confidence intervals were calculated from 2 × 2 contingency tables, with confidence intervals estimated on the log-odds scale. Age was compared between groups with the independent t-test. For proportions in the cleat subgroup, 95% confidence intervals were calculated using the Wilson score method. Findings related to cleat status were treated as exploratory and descriptive because cleat status was undetermined in four bicycle-related cases and the confirmed cleat subgroup was small. In addition, a logistic regression model was fitted with anterior column involvement as the dependent variable and bicycle accident, age (as a continuous variable), and sex as the independent variables, and the same model was fitted with pure anterior column fracture as the dependent variable. Age and sex were selected a priori because they were available for all patients and were clinically plausible confounders. Bone quality and injury energy were not available in the data warehouse and could not be included in the models. These models were used to examine whether the association between bicycle accidents and anterior column involvement remained after adjustment for the available patient factors, and not to identify independent predictors of fracture morphology. Because seven patients contributed two fractures each, the models were repeated after exclusion of the fractures of these seven patients as a sensitivity analysis. A *p*-value of less than 0.05 was regarded as statistically significant for the primary bicycle-versus-other comparisons. All analyses were performed with PASW Statistics for Windows, Version 18.0 (SPSS Inc., Chicago, IL, USA) and Python 3.13 (pandas 3.0.5, SciPy 1.18.1, and statsmodels 0.15.0).

No formal sample size calculation was performed because the study analyzed all eligible patients in the database during the study period.

### 2.6. Use of Generative Artificial Intelligence

Generative artificial intelligence tools were used in the preparation of this study as follows. Claude (Claude Fable 5.1; Anthropic, San Francisco, CA, USA) was used to assist in drafting and language editing of the manuscript, in preparing the flow diagram (Figure 2), in assisting with the schematic annotation of the conceptual illustration of the force pathway presented in Section 4.3, and in writing the Python code for the statistical analysis. ChatGPT (GPT-5; OpenAI, San Francisco, CA, USA) was used to generate the conceptual schematic illustration of the clipless pedal system presented in Figure 1; the authors specified the content, the panel structure, and the labelling of this illustration, and it does not represent any patient, any measurement or any specific commercial product. The underlying clinical image of that illustration was not generated or altered by any artificial intelligence tool. ChatGPT (GPT-5.6 Sol; OpenAI) was used to check the revised manuscript for consistency and language. The study design, the data collection, the review of the medical records and images, the fracture classification, the interpretation of the results, and the conclusions were made by the authors. All outputs of these tools were reviewed and edited by the authors, the statistical results were verified by the authors, and the authors take full responsibility for the content of this publication.

## 3. Results

### 3.1. Overall Cohort and Fracture Distribution

A total of 378 patients with 385 acetabular fractures were analyzed. The mean age of the patients was 52.9 years (standard deviation 15.1; range 13 to 94 years), and 269 patients (71.2%) were men. The distribution according to the Judet–Letournel classification is shown in Table 1. The posterior wall fracture was the most frequent single pattern (83 fractures, 21.6%), followed by the anterior column fracture (70 fractures, 18.1%) and the both-column fracture (52 fractures, 13.5%). Anterior column involvement, as defined above, was present in 154 of 385 fractures (40.0%).

### 3.2. Mechanism of Injury

The distribution of the mechanism of injury among the 378 patients is shown in Table 2. A fall from height was the most frequent mechanism (105 patients, 27.7%). A bicycle accident was the mechanism in 26 patients (6.9%).

### 3.3. Comparison Between Bicycle-Related and Other Fractures

The fracture patterns of the 26 bicycle-related fractures were compared with those of the remaining 359 fractures (Table 3). None of the 26 patients with a bicycle-related injury had bilateral acetabular fractures; therefore, these 26 patients contributed 26 fractures. The 359 comparator fractures were contributed by 352 patients, including the seven patients with bilateral fractures. A pure anterior column fracture was present in 10 of 26 bicycle-related fractures (38.5%) compared with 60 of 359 fractures caused by other mechanisms (16.7%) (odds ratio 3.11, 95% CI 1.35–7.20; *p* = 0.014).

Anterior column involvement was present in 17 of 26 bicycle-related fractures (65.4%) compared with 137 of 359 fractures caused by other mechanisms (38.2%) (odds ratio 3.06, 95% CI 1.33–7.06; *p* = 0.011).

The age and sex distribution of the two groups was similar. At the fracture level, the mean age was 51.0 years (standard deviation 15.6) for the bicycle-related fractures and 53.2 years (standard deviation 15.1) for the fractures caused by other mechanisms (*p* = 0.484), and the proportion of men was 69.2% (18 of 26) and 71.3% (256 of 359), respectively (*p* = 0.824). After adjustment for age and sex, the association between bicycle accident and anterior column involvement remained (adjusted odds ratio 3.02, 95% CI 1.31–6.98; *p* = 0.010). The adjusted odds ratio of bicycle accident for pure anterior column fracture was 3.35 (95% CI 1.43–7.85; *p* = 0.005). After excluding the 14 fractures of the seven patients with bilateral fractures (371 fractures), the adjusted odds ratios of bicycle accident were 3.02 (95% CI 1.30–6.98; *p* = 0.010) for anterior column involvement and 3.19 (95% CI 1.36–7.49; *p* = 0.008) for pure anterior column fracture.

In contrast, posterior wall fractures were less frequent after a bicycle accident (3 of 26, 11.5%) than after other mechanisms (80 of 359, 22.3%), although this difference was not statistically significant (odds ratio 0.45, 95% CI 0.13–1.55; *p* = 0.321).

### 3.4. Characteristics of the Bicycle Accident Group

Among the 26 patients whose acetabular fracture resulted from a bicycle accident, 6 patients (23.1%) sustained the injury in a collision with another vehicle, and the remaining 20 patients sustained the injury in a fall that did not involve another vehicle.

Regarding the use of cleat shoes, 12 patients (46.2%) were confirmed to have been wearing cleat shoes at the time of injury, 10 patients (38.5%) were confirmed not to have been wearing cleat shoes, and in 4 patients (15.4%) the information could not be obtained. Among the 22 patients in whom the information was available, cleat shoes had been worn in 12 (54.5%).

### 3.5. Fractures Sustained While Wearing Cleat Shoes

The demographic and treatment data of the 12 patients who were wearing cleat shoes are shown in Table 4. The mean age was 44.2 years (standard deviation 12.1; range 26 to 66 years). There were 8 men (66.7%) and 4 women (33.3%). The left side was involved in 7 patients (58.3%) and the right side in 5 patients (41.7%). Ten patients (83.3%) were treated operatively, and 2 patients (16.7%) were treated non-operatively.

**Figure 3:**
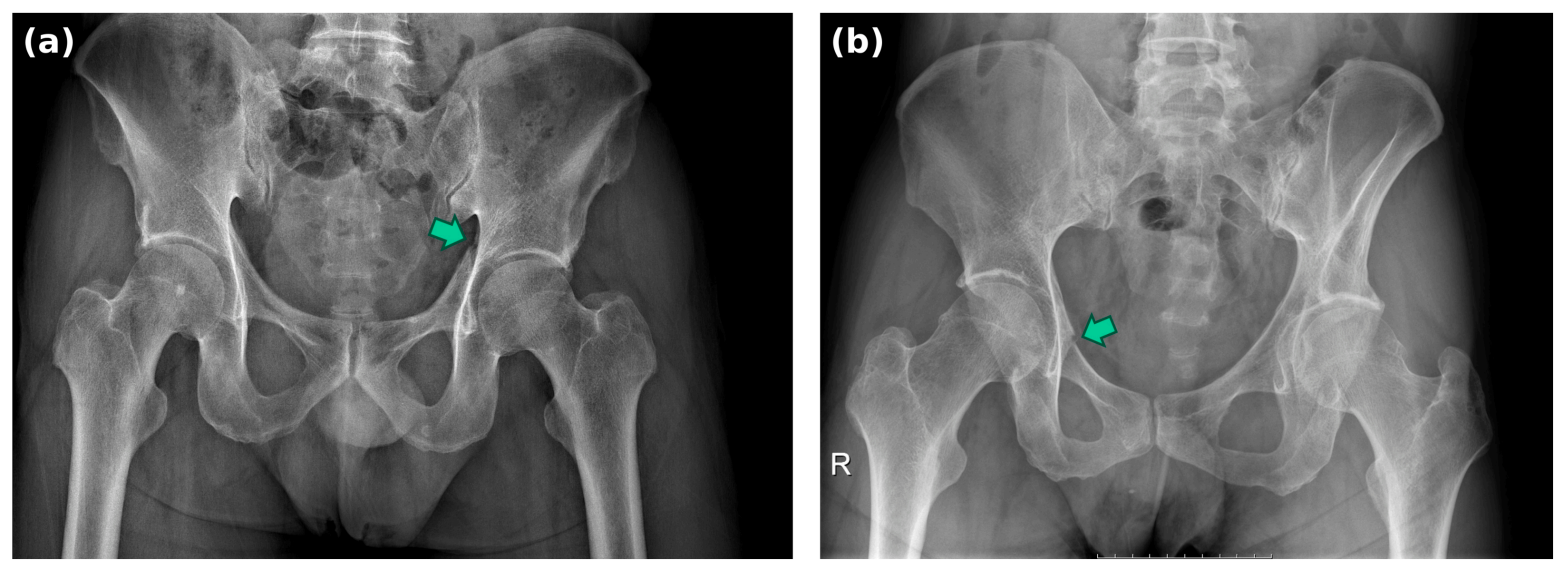
Representative cases of anterior column acetabular fracture sustained in a bicycle accident while wearing cleat shoes. (**a**) Case 1, a 49-year-old man with a left-sided fracture. (**b**) Case 3, a 44-year-old man with a right-sided fracture. The arrow in each panel indicates the interrupted iliopectineal line. R, right side of the patient.

As shown in Table 3, a pure anterior column fracture was the most frequent pattern in this subgroup (7 of 12, 58.3%), followed by anterior column with posterior hemitransverse (2 of 12, 16.7%). Anterior column involvement was present in 10 of 12 fractures (83.3%). Only one posterior wall fracture (8.3%) occurred in this subgroup. Representative cases are shown in Figure 3.

The additional morphological features of these 12 fractures are shown in Table 5. Extension of the fracture line into the acetabular roof was the most frequent feature and was present in 8 fractures (66.7%). Extension to the level of the iliopubic eminence was present in 6 fractures (50.0%), and impaction of the superior dome in 3 fractures (25.0%).

## 4. Discussion

### 4.1. Principal Findings

In this multicenter cohort, 26 of the 378 patients with an acetabular fracture (6.9%) were injured in a bicycle accident. Previous cycling-injury cohorts have reported acetabular fractures within broader fracture populations. In particular, the Swedish Fracture Register identified 179 acetabular fractures among 33,384 bicycle-related fractures [2]. However, that study started from cycling injuries and then described the distribution of fractures. To our knowledge, the present study is the first to start from an acetabular-fracture cohort and to compare Judet–Letournel morphology between bicycle-related fractures and fractures caused by other mechanisms.

Bicycle-related acetabular fractures showed a higher proportion of anterior column involvement. Anterior column involvement was present in almost two-thirds of bicycle-related fractures compared with slightly more than one-third of fractures caused by other mechanisms, whereas posterior wall fractures were less frequent after bicycle accidents. The association remained after adjustment for age and sex, and the adjusted odds ratio was almost the same as the unadjusted odds ratio, consistent with the similar age and sex distributions of the two groups. The result was also unchanged when the patients with bilateral fractures were excluded. However, this comparison has important limitations. The number of bicycle-related fractures was small (n = 26), and the comparison group was heterogeneous and included high-energy mechanisms such as falls from height and motor vehicle accidents. Bone quality and injury energy could not be adjusted for. Therefore, this observation should be interpreted as an association in the distribution of fracture patterns, and not as an independent effect of bicycle injury.

Among riders confirmed to have been wearing cleat shoes, pure anterior column fractures were present in 58.3%, anterior column involvement in 83.3%, and fracture extension into the acetabular roof in 66.7%.

### 4.2. Bicycle Accidents as a Distinct Injury Mechanism

Prior literature specifically focused on cycling-related acetabular morphology is limited. Swart et al. reported four cycling-related acetabular fractures, all involving the anterior column [1]. Broader cycling fracture registries have shown that acetabular fractures are part of the cycling injury spectrum, but they did not describe Judet–Letournel acetabular morphology [2]. Our finding that anterior column involvement was present in 17 of 26 bicycle-related fractures is consistent with the pattern described by Swart et al. and extends that observation to a multicenter acetabular-fracture cohort.

An important difference between our study and the focused case literature is that the earlier reports emphasized diagnostic delay and largely non-operative management [1]. In the present cleat subgroup, 10 of 12 patients (83.3%) underwent operative treatment. However, fracture displacement was not measured, reduction quality was not graded, and clinical and radiographic outcomes after treatment were not collected in this study. Operative indications were also not standardized across the institutions. Therefore, the operative rate should be understood only as a description of the treatment that was given, and it does not show that cleat-related injuries were more severe or that the treatment results were better or worse.

### 4.3. Pathomechanical Considerations

Previous descriptions of acetabular injury mechanics have shown that the fracture pattern produced by force transmitted through the femoral head depends on the hip position at impact, the magnitude of the force, and patient factors such as age and bone quality [10,11]. With direct lateral loading through the greater trochanter, changes in hip rotation and in abduction or adduction can alter the region of acetabular force transmission and the resulting fracture plane [10,11].

A cyclist who falls sideways while the foot remains attached to the pedal may strike the lateral aspect of the hip before protective foot placement occurs. If the constrained lower limb is relatively externally rotated during the fall, the transmitted force may favor anterior acetabular involvement. This is only a proposed pathomechanical explanation for the observed pattern. The actual sequence was not directly measured in the present patients.

**Figure 4:**
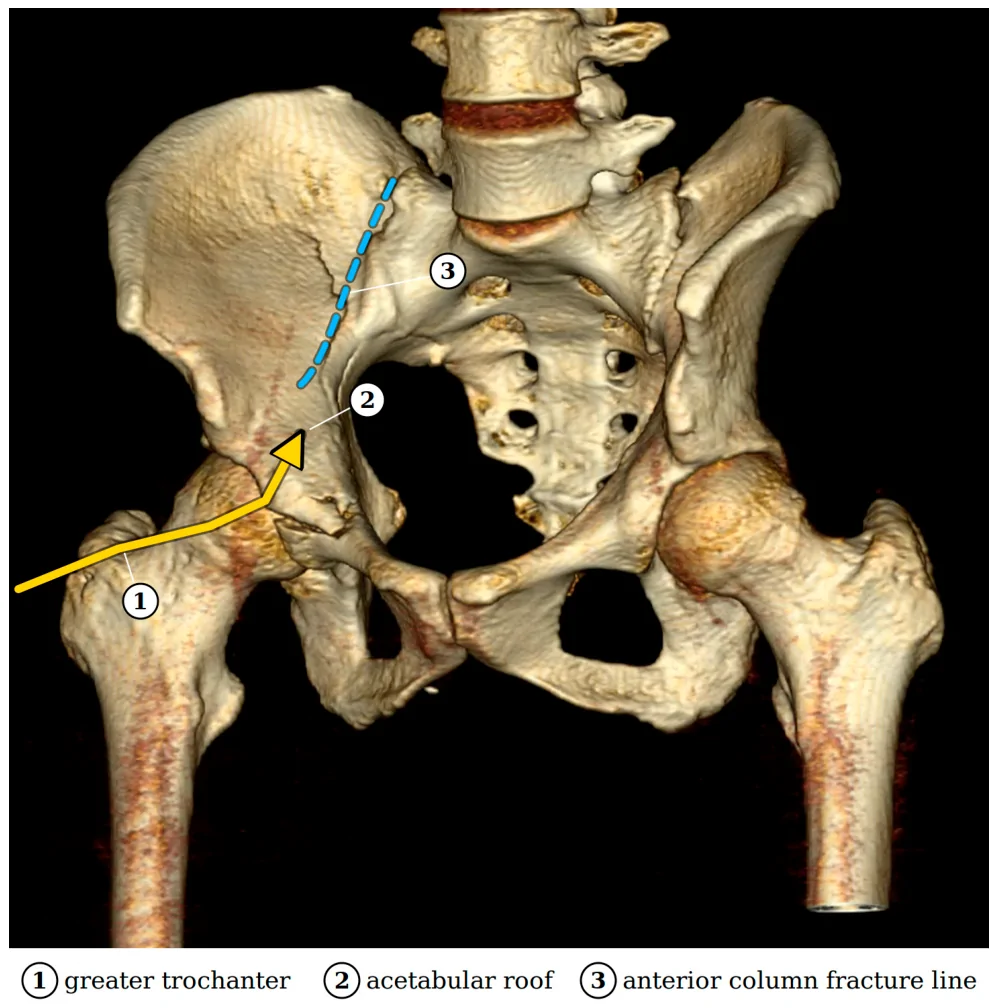
Conceptual illustration of the proposed force pathway, shown on a three-dimensional computed tomography reconstruction of the pelvis of Case 3, a 44-year-old man with a right anterior column fracture sustained in a bicycle accident while wearing cleat shoes. The yellow arrow schematically indicates a hypothesized pathway of force transmission from the greater trochanter (1), through the femoral neck and femoral head, toward the anterosuperior acetabulum (2). The blue dashed line follows the anterior column fracture line (3). This schematic does not represent a measured or reconstructed patient-specific force vector. The right side of the patient is on the left of the image.

This proposed mechanism has similarities to low-energy acetabular fractures in older adults. In geriatric patients, lateral impact can transmit force from the greater trochanter through the femoral neck and head toward the anterior and superior acetabulum, and anterior column or anterior column with posterior hemitransverse patterns are common [6,7] (Figure 4).

The pattern observed in our bicycle-related fractures was similar to the anterior-dominant pattern that is recognized in geriatric patients. This similarity may indicate that lateral impact mechanics contribute in both situations, but it does not prove that the two mechanisms are the same. The additional morphological features recorded in the cleat subgroup were assessed descriptively and were not compared with a matched control group. The pathomechanical explanation proposed above is one possible explanation for the observed pattern. Differences in bone quality, injury energy, and the circumstances of the fall between the bicycle-related patients and the patients injured by other mechanisms may also have contributed to the difference in fracture pattern, and these factors could not be separated in the present study.

### 4.4. The Role of Cleat Shoes

Among the 22 bicycle-related patients for whom cleat status could be determined, 12 patients were wearing cleat shoes at the time of injury, and the anterior column was involved in 10 of these 12 fractures.

The present data cannot show that cleat shoes or clipless pedals increase the risk of acetabular fracture or the probability of a specific fracture pattern. Such a claim would require a reliable denominator for clipless-pedal exposure in the underlying cycling population, and it would also require a considerably larger cohort with complete exposure ascertainment. In addition, cleat status was undetermined in four bicycle-related patients, and the fracture patterns of the confirmed cleat subgroup could not be separated from the effects of age, sex, bone quality, injury energy, the mechanism of the fall, or the involvement of another vehicle. Furthermore, wearing cleat shoes does not confirm that the cleat remained engaged with the pedal at the moment of impact. For these reasons, the cleat-related findings of this study are strictly exploratory and non-causal, and no conclusion about an exposure effect is drawn from the cleat subgroup.

A rider who fails to disengage may be unable to place the foot on the ground in time, and may have less opportunity to reduce the impact before the lateral aspect of the hip strikes the ground. Parker et al. documented this sequence in a club cyclist who could not release his feet from clipless pedals and sustained a direct hip impact with a displaced femoral neck fracture [3]. This is a hypothesis that may explain the anterior-dominant pattern, but it was not tested in the present study. Whether the cleat remained engaged at the moment of impact could not be verified in the present patients. Prospective studies should record the pedal type, the engagement or release of the cleat during the fall, and the cycling exposure at presentation.

### 4.5. Clinical Implications

When a cyclist presents after a fall onto the lateral aspect of the hip and complains of persistent groin or hip pain, an acetabular fracture involving the anterior column should be actively considered. An undisplaced anterior column fracture produces only a subtle change in the iliopectineal line on the anteroposterior radiograph, and Swart et al. emphasized that these fractures are frequently missed on plain radiographs [1]. Computed tomography should be obtained without delay when the clinical suspicion persists despite normal-appearing radiographs.

Because anterior column involvement was frequent, the anterior column, the quadrilateral surface, and the weight-bearing dome should be assessed carefully on preoperative computed tomography. Anterior-based approaches such as the modified Stoppa approach are established options for selected anterior-dominant acetabular fractures [4,6], but the present study was not designed to compare or recommend surgical approaches. In the cleat subgroup, the fracture frequently extended into the acetabular roof, which also supports careful assessment of the weight-bearing dome during preoperative planning.

The anterior-dominant morphology observed in this study may also be relevant to the choice of treatment. In a recent systematic review of elderly patients treated with fixation combined with acute total hip arthroplasty for an acetabular fracture, low-energy trauma from a ground-level fall was the most common mechanism (64%), and the anterior column with posterior hemitransverse pattern was the most prevalent fracture type (30.6%), followed by the both column pattern (15.9%) [12]. In that review, preexisting osteoarthritis, impaction of the acetabular dome or the femoral head, intra-articular comminution, posterior wall involvement, and poor bone quality were the factors considered when the combined procedure was selected instead of fixation alone [12]. In the present study, dome impaction, one of these factors, was present in one quarter of the fractures of the confirmed cleat-shoe users, and the fracture extended into the acetabular roof in two-thirds. The weight-bearing dome should therefore be evaluated carefully on computed tomography before the treatment is decided. However, the bicycle-related patients in this study were younger than the patients in that review (mean age of the cleat subgroup, 44.2 years), and operative treatment was performed in most of them. The present study did not evaluate reduction quality or clinical outcomes, and it cannot recommend one treatment strategy over another.

The findings may also be useful for cyclists. A sideways fall from the bicycle, even without a collision with a vehicle, can result in an acetabular fracture when the rider lands directly on the lateral aspect of the hip. The symptoms may be mild at first, and the plain radiographs may appear normal. Therefore, a cyclist who has persistent groin or hip pain after such a fall should seek medical evaluation rather than assume that the injury is a contusion. For riders who use clipless pedals, awareness of the release mechanism and of the need to disengage early when balance is lost may be useful. However, these suggestions are based on the descriptive findings of this study and on the mechanism described in previous case reports [1,3]. The present study did not establish a causal relationship between clipless pedal use and the fracture pattern, and it does not provide evidence for or against the use of clipless pedals.

### 4.6. Limitations

This study has several limitations that should be considered when the results are interpreted.

First, this was a retrospective study based on a clinical data warehouse, and case identification depended on ICD-10 coding and the completeness of historical electronic records. The search covered 2010–2023, although the integrated Clinical Data Warehouse was implemented in 2019, and variation in historical data integration or documentation quality across the study period cannot be completely excluded. The level of detail regarding injury mechanism was not uniform. In addition, although the comparison between bicycle-related and other fractures was adjusted for age and sex, bone quality and injury energy were not available for the cohort and could not be adjusted for. Residual confounding by these factors may therefore contribute to the observed association between bicycle injury and fracture morphology. The 14-year study period may also include temporal changes in cycling participation, clipless-pedal use, imaging practice and referral patterns.

Second, ascertainment of cleat-shoe use was limited. This information was not routinely recorded in the electronic medical record and remained undetermined in 4 of 26 bicycle-related patients. When medical records did not contain the information, telephone interviews were conducted between March and July 2024, so that the recall intervals were variable and recall bias was possible. Furthermore, wearing cleat shoes does not prove that the cleat remained engaged at impact, and disengagement during the fall could not be verified.

Third, the confirmed cleat subgroup included only 12 patients. Confidence intervals around the morphological proportions are therefore wide, and the study was not designed or powered to establish an exposure effect of clipless pedals. Additional features such as acetabular roof extension, iliopubic eminence extension and dome impaction were evaluated descriptively in this subgroup without a matched comparator. In addition, 6 of the 26 bicycle-related injuries involved a collision with another vehicle. These cases were retained because the exposure was defined as bicycle involvement at the time of injury. However, collision-related injuries may involve force vectors and injury energies that differ from those of simple lateral falls, and this introduces heterogeneity in the injury mechanism.

Fourth, there were several radiographic and analytic limitations. Fracture displacement, including maximal gap and step-off, was not quantified, and operative indications were determined by the treating surgeons rather than by a predefined protocol. The fractures were classified by one surgeon and verified by a second surgeon, but the two surgeons did not classify the fractures independently, and interobserver agreement was not measured. The Judet–Letournel system is widely used, but its reproducibility varies with observer experience and imaging interpretation [13], and some fractures might have been classified differently by other observers. In addition, although the imaging modalities were the same at the six hospitals, the acquisition parameters, such as the computed tomography scanner and the slice thickness, were not standardized across the hospitals or across the study period. Seven patients in the overall cohort had bilateral acetabular fractures, which creates a small degree of non-independence in the fracture-level analyses; a sensitivity analysis that excluded these patients gave the same results. None of the 26 bicycle-related patients had bilateral fractures.

Fifth, no clinical or longitudinal radiographic outcomes were collected. Reduction quality, functional scores, post-traumatic osteoarthritis, avascular necrosis and conversion to total hip arthroplasty were not evaluated. Several other variables were also not systematically available, namely body mass index, bone mineral density, cycling speed and experience, bicycle type, and the detailed circumstances of the fall. Road cycling has been associated with lower bone mineral density at the lumbar spine and hip in some adult cyclists [14], but the contribution of bone quality to the present fractures could not be examined.

The limitations described above indicate the design of a future study. A prospective multicenter registry of cycling-related pelvic and acetabular injuries should record, at presentation, the detailed mechanism of the fall, the body position and the side of impact, the riding speed, the pedal type, whether the cleat was engaged or released at the moment of the accident, whether another vehicle was involved, the body mass index and the bone mineral density. The fracture classification should be performed independently by two or more observers with a prespecified interobserver reliability assessment, and the displacement, the reduction quality and the clinical outcomes should be measured with standard methods. Such a study would allow the relationship between the mechanism of a bicycle accident, the use of clipless pedals and the acetabular fracture pattern to be analyzed with adjustment for the relevant confounders.

### 4.7. Strengths

The main strength of this study is the multicenter cohort of 385 acetabular fractures collected from six hospitals of a single university network through an integrated clinical data warehouse [8,9], which provides a denominator large enough to estimate the contribution of bicycle accidents. In addition, this is the first report that compares the fracture pattern of bicycle-related acetabular fractures with that of other injury mechanisms.

## 5. Conclusions

In this multicenter cohort of 378 patients with 385 acetabular fractures, 26 patients (6.9%) sustained the fracture in a bicycle accident. Bicycle-related fractures were associated with a higher proportion of anterior column involvement than fractures caused by other mechanisms, and this association remained after adjustment for age and sex. Bone quality and injury energy could not be adjusted for, and the number of bicycle-related fractures was small, so that the association should not be interpreted as a causal effect. Among 12 patients confirmed to have been wearing cleat shoes, anterior column patterns and acetabular roof extension were common; however, these observations are descriptive and exploratory and do not establish an independent effect of clipless pedals. This study described the fracture patterns only. Fracture displacement, reduction quality and clinical outcomes were not evaluated, and the findings do not provide information on the results of treatment. Clinicians should consider an anterior column acetabular fracture when persistent hip or groin pain follows a cycling-related lateral fall, particularly when plain radiographs are unrevealing. Prospective studies with systematic collection of the accident mechanism, the pedal exposure and the patient factors are needed before any conclusion about the role of clipless pedals can be drawn.

## Figures and Tables

**Table 1:** Distribution of 385 acetabular fractures according to the Judet–Letournel classification.

Fracture Type	n (%)
Anterior wall	50 (13.0)
Anterior column	70 (18.1)
Posterior wall	83 (21.6)
Posterior column	14 (3.6)
Posterior column with posterior wall	11 (2.9)
Transverse	20 (5.2)
Transverse with posterior wall	21 (5.5)
T-shaped	32 (8.3)
Anterior column with posterior hemitransverse	32 (8.3)
Both column	52 (13.5)
Total	385 (100.0)

**Table 2:** Mechanism of injury in 378 patients with an acetabular fracture.

Mechanism	n (%)
Fall from height	105 (27.7)
In-car traffic accident	76 (20.1)
Slip down	69 (18.3)
Motorcycle accident	48 (12.7)
Pedestrian traffic accident	36 (9.5)
Bicycle accident	26 (6.9)
Other	18 (4.8)
Total	378 (100.0)

**Table 3:** Judet–Letournel classification of the 26 bicycle-related acetabular fractures, and of the subgroup of 12 fractures sustained while wearing cleat shoes.

Fracture Type	All Bicycle Accidents, n = 26 (%)	With Cleat Shoes, n = 12 (%)
Anterior wall	2 (7.7)	0 (0)
Anterior column	10 (38.5)	7 (58.3)
Posterior wall	3 (11.5)	1 (8.3)
Posterior column	1 (3.8)	0 (0)
Posterior column with posterior wall	0 (0)	0 (0)
Transverse	0 (0)	0 (0)
Transverse with posterior wall	1 (3.8)	0 (0)
T-shaped	2 (7.7)	1 (8.3)
Anterior column with posterior hemitransverse	3 (11.5)	2 (16.7)
Both column	4 (15.4)	1 (8.3)
Total	26 (100.0)	12 (100.0)

**Table 4:** Demographic and treatment data of the 12 patients who sustained an acetabular fracture in a bicycle accident while wearing cleat shoes.

Case	Age (Years)	Sex	Side	Treatment	Judet–Letournel Classification
1	49	M	Left	Operative	Anterior column
2	39	F	Right	Operative	Anterior column
3	44	M	Right	Operative	Anterior column
4	49	M	Right	Operative	Anterior column with posterior hemitransverse
5	60	M	Left	Non-operative	Anterior column
6	26	M	Left	Operative	T-shaped
7	43	F	Left	Operative	Anterior column
8	66	M	Right	Operative	Both column
9	54	M	Left	Operative	Anterior column
10	29	F	Left	Operative	Anterior column with posterior hemitransverse
11	33	M	Left	Operative	Posterior wall
12	38	F	Right	Non-operative	Anterior column

**Table 5:** Morphological features of the 12 acetabular fractures sustained while wearing cleat shoes. The features are not mutually exclusive.

Feature	n (%)	95% CI (%)
Extension to the acetabular roof	8 (66.7)	39.1–86.2
Extension to the iliopubic eminence	6 (50.0)	25.4–74.6
Dome impaction	3 (25.0)	8.9–53.2
Posterior hemitransverse component	2 (16.7)	4.7–44.8
Both column pattern	1 (8.3)	1.5–35.4

CI, confidence interval.

## Data Availability

The data presented in this study are available on reasonable request from the corresponding author. The data are not publicly available because of institutional privacy restrictions.
